# Juvenile Dermatomyositis in Pregnancy

**DOI:** 10.1155/2013/890107

**Published:** 2013-04-15

**Authors:** Anthony Emeka Madu, Edwin Omih, Elaine Baguley, Stephen W. Lindow

**Affiliations:** ^1^Women and Children Hospital, Hull Royal Infirmary, Anlaby Road, Hull HU3 2JZ, UK; ^2^Department of Rheumatology, Hull Royal Infirmary, Anlaby Road, Hull HU3 2JZ, UK

## Abstract

Juvenile dermatomyositis has variable clinical presentations both in and outside of pregnancy. A literature review indicated that optimal maternal and fetal outcomes can be anticipated when the pregnancy is undertaken while the disease is in remission. Poorer outcomes are associated with flare-up of the disease in early pregnancy compared with exacerbation in the second or third trimester, when fetal prognosis is usually good. We present a case of JDM in pregnancy with disease exacerbation late in pregnancy and review of the relevant literature.

## 1. Introduction

Juvenile dermatomyositis (JDM) is presumed to be idiopathic inflammatory myopathy, possibly with a genetic component presumed to be due to autoimmune dysfunction. Circulating immune complexes are present causing vasculitis resulting in mainly skin and muscle manifestations among other complications. It has a variable progression and variable involvement of skin and muscle. Worldwide incidence of JDM is approximately 2-3 cases per million children per year with some differences between ethnic groups with a female preponderance of 2 : 1 [1]. It is usually triggered by a condition that evokes immune system activity that does not resolve or abate. Common triggers for JDM include vaccinations, infections, injuries, and sunburn. 

The vasculopathy of JDM is manifested predominantly in two ways: distinctive unique pinkish, heliotrope rash affecting the face, eyelids, hands, and sometimes the skin over the joints, including the knuckles, knees, and elbows. Some children develop calcium deposits (calcinosis) under the skin [1, 2]. The second symptom of vasculopathy is muscle inflammation that is manifested as fatigue, clumsiness, not keeping up physically with peers, and eventually inability to perform physical tasks. Other signs include falling, dysphonia, and dysphagia. The muscle weakness mimics that of muscular dystrophy or other muscle disease. Some patients develop contractures when proximal muscles are first affected. About half of children with JDM are stated to have pain in their muscles [2]. JDM can also affect the heart, lungs, and oesophagus. Given the tendency of this disease to affect more young females compared to males, the interaction of the disease and pregnancy is not well described. 

## 2. Case History

A 22-year-old woman presented in her first pregnancy and booked at 10-week gestation. She was a known case of JDM which was diagnosed in childhood and was treated with a maintenance dose of prednisolone 5 mg on alternate days before pregnancy. In addition to the typical skin lesions as a child, she had also presented with difficulty in swallowing. However a barium swallow performed by paediatric radiologist was normal. This difficulty in swallowing resolved in early childhood. She had occasional palpitations and dizziness as a child and as a teenager. An echocardiogram revealed slightly reduced left ventricular ejection fraction of 52%. A subsequent echocardiogram revealed episodes of sinus tachycardia only. There was no evidence of lung involvement following radiological examination during investigation in her childhood and teenage years. 

The patient had been taking folic acid 5 mg daily for 4 years prior to conception. She was found to be allergic to azathioprine which gave her rash; hence it was considered inappropriate to continue to use this medication. Her body mass index (BMI) was 18.1 (weight 50.5 kg and height 167 cm) on booking. She had stopped the combined oral contraceptive pill and methotrexate (12.5 mg daily) about 2 months before conception as advised by her general practitioner during prepregnancy consultations. She had also stopped hydroxychloroquine (200 mg twice daily) about 5 weeks into her pregnancy. Routine maternal antenatal screen was normal; she was group A rhesus negative with no maternal antibodies. She was managed jointly by a multidisciplinary team which included an obstetrician, paediatrician, rheumatologist, and anaesthetist. 

Routine fetal anomaly scan at 20-week gestation was normal. She had normal liquor volume and a normal umbilical artery Doppler analysis at 34, 36, 37, and 38 weeks gestation. At 28-week gestation she had iron-deficiency anaemia with haemoglobin (Hb) of 9.7 g/dL and was given ferrous sulphate tablets 200 mg thrice daily. At 36 weeks gestation, her Hb level improved to 11.9 g/dL and at 37-week it was 12.1 g/dL. 

At about 36-week gestation, she had an anaesthetic review and the plan was that she could have regional anaesthesia in labour. However, it was noted she was at risk of increased sensitivity to muscle relaxants. 

Just after 39-week gestation, she presented with flare-up of her skin lesions, sore skin, sore face, reddish scaling of inner part of her lips, and bluish scaling-outer part of her lips. She was then induced with vaginal prostaglandins, had spontaneous membrane rupture, progressed in labour, and subsequently had epidural analgesia and syntocinon augmentation. She delivered a healthy female baby weighing 2790 g, with Apgar score of 9 at 1 minute and 9 at 5 minutes. Third stage of labour was actively managed with intravenous oxytocin and subsequently intramuscular syntometrine (mixture of oxytocin, 5 IU and ergometrine, 500 mcg). After delivery, the plan was to give her prednisolone in the following sequence: 30 mg daily for 1 week, 20 mg daily for 1 week, 15 mg daily for 1 week, 10 mg daily for 1 week, and then continue on 5 mg alternate days. There was no record of hydrocortisone cover in labour, and she did not have any maternal collapse or hypotension intrapartum or postpartum. She was discharged after 2 days. Her baby was doing very well upon discharge with a community paediatric followup at visits home.

She presented to the rheumatologist as an emergency 10 days following delivery complaining of feeling unwell, tired, poor appetite, increased muscle weakness, and weight loss. Following clinical assessment by the rheumatologist, the latter concluded that the dose of prednisolone after delivery may have been too much. Her prednisolone was therefore reduced from 30 mg daily to 2.5 mg daily. This resulted in improvement of her clinical status. However, when reviewed at the 6 weeks postnatal clinic, her skin was still active and she was on prednisolone 2.5 mg daily. Other maternal assessments were satisfactory. Her baby was assessed again after 6 weeks' post delivery in the paediatric clinic and observations were satisfactory. Her care was then transferred back to the rheumatologist. Mother and baby had continued to do well.

## 3. Discussion

Gutiéarrez et al. [3] published a case series of 18 women with polymyositis/dermatomyositis describing a 55% increase in the rate of fetal loss and 50% of pregnancies ended prematurely. There was no correlation between disease activity and fetal loss. The authors concluded that polymyositis and dermatomyositis should be considered high risk for both mother and baby. Our case was also classed as a high-risk pregnancy with multidisciplinary care involving obstetric consultant, midwives, rheumatologist, anaesthetists, and her general practitioner.

By 1992 only 19 cases of JDM have been published according to Pinheiro Gda et al. [4] who published the first case of JDM in pregnancy that ended with emergency caesarean section at 37-week gestation and at 8-month follow-up mother and baby were doing well. Our case fortunately in contrast ended in normal delivery after induction of labour. 

Silva et al. [5] published a more detailed series of pregnancy in 28 women with dermatomyositis and polymyositis and concluded that fetal prognosis reflects the level of maternal disease. The more active the myositis during pregnancy, the greater the chances of fetal loss. In our case, maternal disease was relatively inactive during much of the pregnancy but for the skin flare-up at about 39-week gestation. At that stage, a decision was made to induce and deliver the baby for the sake of mother and her baby.

The variable nature of the clinical presentation in pregnancy support caution was exemplified in the case reported by Nozaki et al. [6]. In this case, respiratory muscle weakness with CO_2_ narcosis in pregnancy necessitated treatment with intravenous immunoglobulin therapy after delivery which resulted in remission of the disease. Our case, however, did not present with any respiratory complications in pregnancy.

Detailed search in PUBMED to date yielded only 93 citations suggesting the rarity of the problem. Some of the 93 reviewed citations were JDM and other related inflammatory autoimmune conditions, some of which were reported in combination with JDM.

Our case was that of a young woman who was in remission before her conception and was on prepregnancy maintenance dose of prednisolone while she was pregnant. This dose was able to control her disease until a flare-up just after 39 weeks of gestation. This skin flare-up necessitated intervention in the form of induction of labour and delivery of a female baby weighing only 2790 g compared to average weight of a female baby at about 40-week gestation which is about 3258 g [7]. JDM, its treatment, and, in general, autoimmune diseases and their management in pregnancy are associated with fetal growth restriction and lower birth weight.

According to Chopra et al. [8], exacerbation in the second and third trimester pregnancy rather than first trimester has been associated with better perinatal outcomes. This is consistent with the clinical findings in our case. There was only one exacerbation at 39-week gestation and the outcome was relatively good compared to some of the cases we reviewed and referenced which ended up in more adverse maternal and fetal outcomes. 

The pharmacological management was steroid based as the patient was allergic to Azathioprine. There is little evidence that azathioprine is teratogenic in pregnancy but some reports have implicated it in causing premature births, low birth weight (in combination with corticosteroids), and spontaneous miscarriage following maternal and paternal exposure. However, this drug is recommended in pregnancy if the benefits outweigh the risks according to Clinical Effectiveness Unit Guidance (CEU) of the Faculty of Sexual and Reproductive Health [9]. There was no records of the patient been on low-dose aspirin both before pregnancy and during the pregnancy. Based on the proven benefit of low-dose aspirin from randomized controlled trials and systematic reviews, low-dose aspirin has been used during pregnancy to reduce the risk of having a premature birth in women who have several risk factors for premature births. This includes previous premature birth or preterm labour, diabetes, high blood pressure, vascular disease or complication, previous early-onset preeclampsia before 32-week gestation, and antiphospholipid syndrome. It is possible that this patient may have benefited from low-dose aspirin during pregnancy.

Methotrexate was stopped at about 2 months prior to conception while hydroxychloroquine was stopped at about 5-week gestation to reduce the risk of teratogenicity. It is also recommended that these drugs be avoided during breastfeeding. 

The benefit of use of corticosteroids is stated to outweigh the risks. Betamethasone and dexamethasone both cross placenta readily; however, as much as 88% of prednisolone is inactivated as it crosses the placenta [9], thus reducing the amount of drug that gets to the fetal circulation. Thus prolonged use of prednisolone rather than short-term use can cause intrauterine growth restriction which was seen in our case. 

This case is different in its presentation, its prepregnancy, antenatal, peripartum, and postpartum care. Disease exacerbation in pregnancy only occurred very late in third trimester and ended in normal delivery without operative intervention. There were no significant perinatal morbidity and mortality.

## 4. Conclusion

In conclusion, our case was managed with maintenance dose of prednisolone. She only had one disease flare-up at term-necessitated delivery and postpartum steroid treatment. The importance of maternal and fetal clinical and obstetrical vigilance is also stressed to improved maternal and fetal outcomes. The mother should be monitored for disease exacerbations and the fetus monitored for growth restriction and features of fetal compromise. It is the authors' view that randomized controlled trials and systematic reviews are needed to clearly define the best or most effective and evidence-based treatment options for these group of patients so as to guide clinicians in the care of these women. For example, randomised controlled trials can be organised by screening and allocating these patients randomly to receive various interventions like various elements of maternity care or even medications like steroid (prednisolone) alone steroid combined with azathioprine, or azathioprine alone. Various units and regional centres in the UK and overseas can integrate their pool of patients and set specific uniform controls before such trial. Various randomised control trials on a similar subject can then be systematically reviewed and analysed in a meta-analysis to get level 1++ evidence. 
